# The Synthesis of Kynurenic Acid in Mammals: An Updated Kynurenine Aminotransferase Structural KATalogue

**DOI:** 10.3389/fmolb.2019.00007

**Published:** 2019-02-27

**Authors:** Franca Rossi, Riccardo Miggiano, Davide M. Ferraris, Menico Rizzi

**Affiliations:** Biochemistry and Biocrystallography Unit, DSF-Dipartimento di Scienze del Farmaco, University of Piemonte Orientale, Novara, Italy

**Keywords:** kynurenine pathway, kynurenic acid, kynurenine aminotransferase, PLP enzyme, crystal structure

## Abstract

Kynurenic acid (KYNA) is a bioactive compound that is produced along the kynurenine pathway (KP) during tryptophan degradation. In a few decades, KYNA shifted from being regarded a poorly characterized by-product of the KP to being considered a main player in many aspects of mammalian physiology, including the control of glutamatergic and cholinergic synaptic transmission, and the coordination of immunomodulation. The renewed attention being paid to the study of KYNA homeostasis is justified by the discovery of selective and potent inhibitors of kynurenine aminotransferase II, which is considered the main enzyme responsible for KYNA synthesis in the mammalian brain. Since abnormally high KYNA levels in the central nervous system have been associated with schizophrenia and cognitive impairment, these inhibitors promise the development of novel anti-psychotic and pro-cognitive drugs. Here, we summarize the currently available structural information on human and rodent kynurenine aminotransferases (KATs) as the result of global efforts aimed at describing the full complement of mammalian isozymes. These studies highlight peculiar features of KATs that can be exploited for the development of isozyme-specific inhibitors. Together with the optimization of biochemical assays to measure individual KAT activities in complex samples, this wealth of knowledge will continue to foster the identification and rational design of brain penetrant small molecules to attenuate KYNA synthesis, i.e., molecules capable of lowering KYNA levels without exposing the brain to the harmful withdrawal of KYNA-dependent neuroprotective actions.

## Introduction

**Figure 1:**
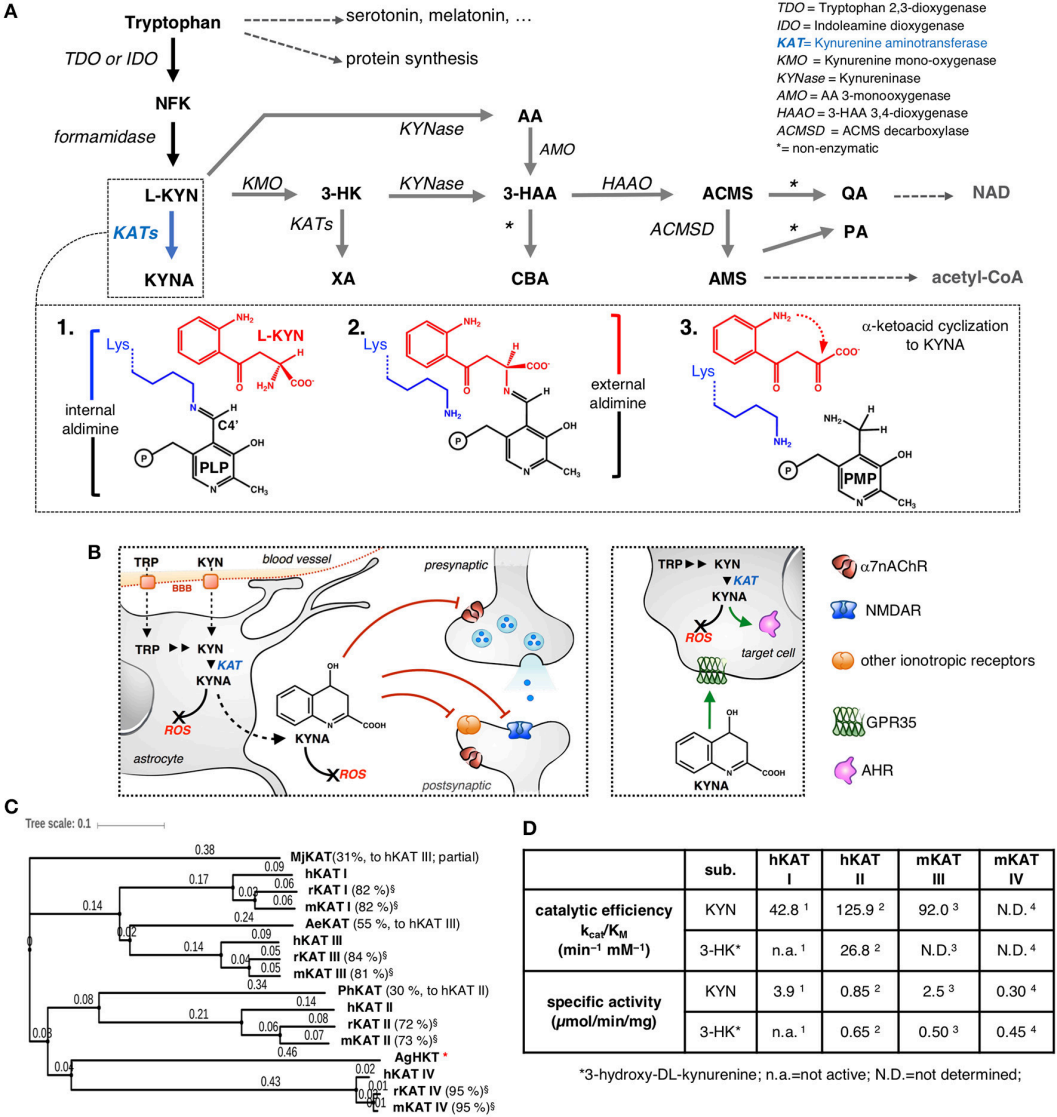
The kynurenine pathway with a focus on KYNA synthesis. **(A)** NFK, N-formylkynurenine; L-KYN, L-kynurenine; KYNA, kynurenic acid; 3-HK, 3-hydroxykynurenine; XA, xanthurenic acid; AA, anthranilic acid; 3-HAA, 3-hydroxyanthranilic acid; ACMS, 2-amino-3-carboxymuconate-6-semialdehyde; AMS, 2-aminomuconate-6-semialdehyde; PA, picolinic acid; CBA, cinnabarinic acid; QA, quinolinic acid; NAD, nicotinamide adenine dinucleotide. *Inset:* **1**. At the beginning of the transamination reaction, the catalytic lysine of KAT is bound to the PLP molecule. **2**. The α-amino group of L-KYN substitutes for Lys and binds to the cofactor, forming an external aldimine intermediate. **3**. At the end of the first half-reaction, the resulting α-keto acid undergoes an intramolecular condensation reaction, releasing the final product, KYNA, and leaving the cofactor in the pyridoxamine phosphate (PMP) form, then, an α-keto acid is required to bring the enzyme back to its initial state through a series of equivalent reversed reactions (not shown). **(B)** The multiplicity of KYNA actions inside and outside the CNS. KYNA acts as an antagonist of specific receptors both at the synapses and at extra-synaptic sites (only selected targets are shown); KYNA possesses immunomodulatory properties mainly by behaving as an agonist/ligand of GPR35 and AHR. KYNA participates in the direct scavenging of ROS; BBB, blood-brain barrier. **(C)** Analysis of protein sequence similarity among different KATs, including representatives of non-mammalian KATs (Mj, *Methanocaldococcus jannaschii*; Ph, *Pyrococcus horikoshii*; Ae, *Aedes aegypti*; Ag, *Anopheles gambiae*; HKT, 3-hydroxykynurenine aminotransferase). The values in brackets refer to the percentage sequence identity to the corresponding human isoenzyme; ^*^no significant sequence identity. The phylogenetic tree was generated by iTOL (Letunic and Bork, 2016). **(D)** Comparison of selected kinetic constants for the transformation of KYN to KYNA and 3-HK to XA catalyzed by the indicated KATs; (Han et al., 2004, 2008a, 2009a, 2010a).

In mammals, approximately 95% of the essential amino acid L-tryptophan that is not used for protein synthesis is metabolized through the kynurenine pathway (KP). Research interests in the KP find a unifying rationale in the ever-increasing demonstrations that the majority of the compounds formed along the pathway play a role in modulating fundamental aspects of biology (Schwarcz et al., 2012; Stone et al., 2013; Schwarcz and Stone, 2017). The KP is a complex catabolic cascade consisting of a multi-step main branch and several lateral arms (Figure 1A). The relative abundance of the molecules generated through the KP in peripheral tissues and inside the central nervous system (CNS) is thought to be governed at different interdependent levels (Badawy, 2017). The whole complement of enzymes operating in the KP is present in the CNS, where the bioactive kynurenines produce multiple and often antithetical effects, including control of synaptic transmission, hyper-stimulation or hypo-functioning of receptor-mediated signaling, and direct excitotoxicity or neuroprotection (Ruddick et al., 2006). In particular, kynurenic acid (KYNA) is considered a “Janus-faced” compound in brain physiology (Rózsa et al., 2008; Wirthgen et al., 2018). In fact, KYNA limits the neurotoxicity associated with the action of excitatory amino acids (Birch et al., 1988) by acting as an endogenous antagonist at the glycine co-agonist site of the *N*-methyl-D-aspartate receptor (NMDAR). In addition, KYNA non-competitively antagonizes the α7-nicotinic acetylcholine receptor (α7nAChR) (Alkondon et al., 2004; Albuquerque and Schwarcz, 2013), modulating important neurophysiological processes. Moreover, KYNA plays a role as a direct reactive oxygen species (ROS) scavenger. On the other hand, abnormally high KYNA levels have been detected in biological samples from patients with schizophrenia (Erhardt et al., 2007; Sathyasaikumar et al., 2011), and the pharmacological elevation of KYNA concentrations in the CNS correlates with cognitive deficits (Chess et al., 2007). These observations point to KYNA as an important player in the onset and progression of neurological and psychiatric diseases that are associated with impaired glutamatergic and/or cholinergic neurotransmission (Stone and Darlington, 2013; Fujigaki et al., 2017). Moreover, KYNA is an agonist of the broadly expressed G protein-coupled receptor 35 (GPR35) (Wang et al., 2006) and the aryl hydrocarbon receptor (AhR) (DiNatale et al., 2010), which are involved in immunomodulation processes (Wirthgen et al., 2018). Taken as a whole, these studies disclose the multiplicity of biological actions of KYNA inside and outside the CNS (Figure 1B).

KYNA cannot easily cross the blood-brain barrier. For this reason, the local concentration of the compound is thought to mainly depend on (i) the absolute availability of tryptophan and/or L-kynurenine (KYN), (ii) the competition that exists between the kynurenine monooxygenase/kynureninase-dependent branches of the catabolic KP cascade (gray arrows in Figure 1A) and the direct transformation of KYN to KYNA, and (iii) the activity of KYNA-synthesizing aminotransferases (Schwarcz et al., 2012). KATs catalyze the irreversible transamination of KYN to KYNA and 3-hydroxykynurenine (3-HK) to xanthurenic acid (XA), thus controlling the routing of KYN and 3-HK to their corresponding branches of the KP (blue arrows in Figure 1A). KAT-dependent catalysis proceeds through a pyridoxal-5'-phosphate (PLP)-dependent transamination reaction, which has been studied in great detail (Bellocchi et al., 2009). More recently, a pathway for the non-enzymatic production of KYNA has been described and shown to be based on the spontaneous oxidation of KYN (Blanco Ayala et al., 2015). At present, information about the contribution of this mechanism to overall KYNA synthesis is scarce, although this pathway could represent a significant source of the molecule under specific circumstances (Ramos-Chávez et al., 2018). Inside the KP, the conversion of KYN to KYNA is the reaction for which the highest number of isozymes have been identified and arbitrarily named kynurenine aminotransferases (KATs). Human and rodent genomes encode four aminotransferases that have been demonstrated to be capable of using KYN as an amino group donor during the first half-reaction (Figure 1A, inset): KAT I (also known as glutamine transaminase K or cysteine conjugate beta-lyase), KAT II (also known as α-aminoadipate aminotransferase), KAT III (also known as glutamine transaminase L), and KAT IV (the mitochondrial aspartate aminotransferase). The interspecies comparison of each KAT reveals a high degree of primary sequence conservation (Yu et al., 2006), with KAT IV representing the most phylogenetically conserved among the four mammalian isozymes (Figure 1C). Conversely, the alignment of the primary sequences of the four KATs from a single species shows that KAT II and KAT IV are the most divergent isozymes, most likely as a consequence of their peculiar features and the different roles played by the N-terminal regions of the two proteins (Han et al., 2010b). Here, we will refer to human, mouse and rat KATs as hKATs, mKATs, and rKATs, respectively. As reviewed in great detail elsewhere (Han et al., 2010a,b; Passera et al., 2011), mammalian KATs are differentially expressed during ontogenesis and display peculiar tissue distribution profiles and sensitivity to the action of endogenous and exogenous modulators. In particular, although the four isozymes possess overlapping biochemical properties, they differ considerably in terms of substrate specificity as well as specific activity and catalytic efficiency toward KYN and 3-HK (Figure 1D). However, it must be noted that a full interspecies comparison of the catalytic properties and enzyme kinetics of KATs is precluded in part by the lack of uniform biochemical assay conditions for the characterization of the individual isozymes.

## Mammalian KAT Structures: *leitmotiv* And Unique Traits

The crystal structures of hKAT I (Rossi et al., 2004; Han et al., 2009b; Nadvi et al., 2017), hKAT II (Han et al., 2008a,b; Rossi et al., 2008a, 2010; Dounay et al., 2012, 2013; Tuttle et al., 2012; Nematollahi et al., 2016a), mKAT III (Han et al., 2009a; Wlodawer et al., 2018), and mKAT IV (Han et al., 2011) in their holo-forms, in different ligand-bound states and in complex with inhibitors, enormously expanded the ability to identify the structural determinants that are the basis for the common features and unique traits displayed by each KAT. More recently, the structure of the apo-form of mature human mitochondrial aspartate aminotransferase was solved (Jiang et al., 2016), however, considering the high percentage of sequence identity between human and mouse KAT IV (95%) and their structural conservation (root mean square deviation = 0.49 Å), only the murine isozyme will be discussed.

**Figure 2:**
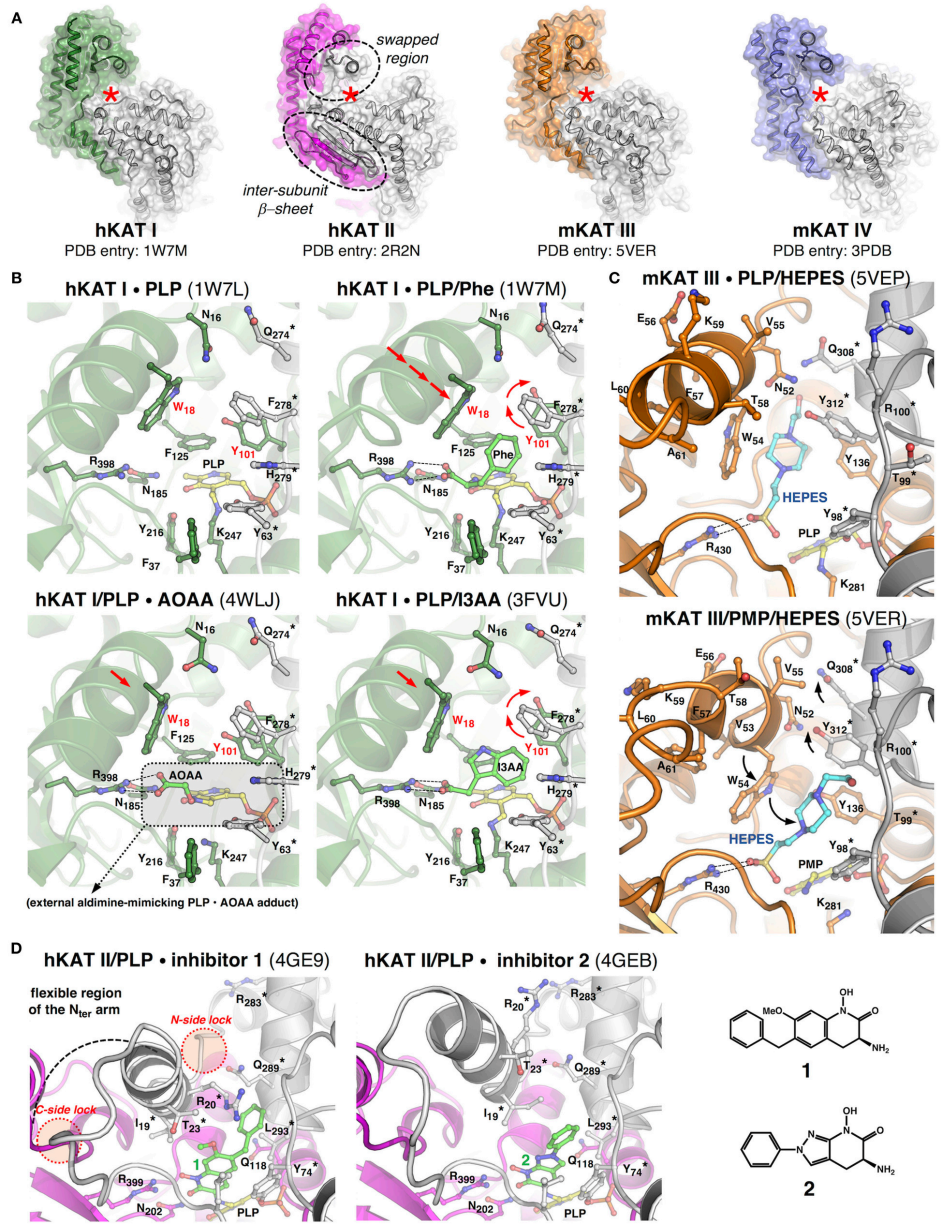
Structural features and properties of mammalian KATs. **(A)** In each KAT dimer, the “A” subunit appears in color, the “B” subunit appears in gray, and the red star labels one of the two identical active sites, the dotted circles frame the peculiar structural features of hKAT II. **(B)** Close-ups of the catalytic cavity of hKAT I in different ligand-bound states. **(C)** The active site of mKAT III in complex with HEPES, which adopts two alternative conformations. **(D)** Zoomed views of the hKAT II active site in complex with two irreversible inhibitors (**1** and **2**). In each image, the protein backbone is depicted as a cartoon, the selected residue side chain is depicted as a ball-and-stick model, the asterisk labels residues belonging to one subunit of the dimer, and the arrows indicate the major rearrangements discussed in the text. The PDB codes appear in brackets. As a matter of clarity, the images, which correspond to optimally superimposed structures, are presented side by side. The figures have been generated by PyMol (www.pymol.org).

All of the KATs that have been studied thus far form homodimers both in solution and in their crystalline state (Figure 2A). This dimerization is required to build up two identical catalytic cavities, each hosting a co-factor molecule, that are located at the interdomain interface in each subunit and at the intersubunit interface in the dimer. Across-species comparison of KAT structures reveals the high degree of conservation of the monomer architecture, which consists of an N-terminal arm, a small domain and a large domain. As a general rule, the N-terminal arm is a crucial element in aminotransferases; it participates in the proper assembly of the functional dimer, controls enzyme subcellular localization, and regulates substrate access to the active site. The globular domains host the majority of the residues that are required for PLP co-factor binding and reactivity, and shaping the ligand binding cavity (Jansonius, 1998). The comparison of representatives of each KAT isozyme reveals peculiar features that characterize the ligand binding site architecture, the mode of dimer assembly, and remarkable differences in the conformational changes that accompany catalysis.

The structures of hKAT I in its PLP-bound pre-reaction state and in complex with PMP at the end of the first half-reaction (PDB entries: 1W7L and 1W7N, respectively) (Rossi et al., 2004) show that catalysis does not significantly alter the overall positioning of the cofactor inside the catalytic cavity, and this holds true for all KATs. Information about ligand binding can be obtained by comparing the structures of hKAT I in complex with phenylalanine (an amino group donor substrate) (PDB entry: 1W7M) (Rossi et al., 2004) or with the inhibitors indole-3-acetic acid (I3AA; PDB entry: 3FVU) (Han et al., 2009b) and 3-amino-oxyacetic acid (AOAA; PDB entry: 4WLJ) (Nadvi et al., 2017; Figure 2B). In the latter structure, a covalent PLP-AOAA adduct is observed, representing a snapshot along the catalytic cycle that catches the cofactor in a state mimicking an external aldimine. These analyses highlight the role of the coordinated action of the strictly conserved arginine and asparagine residues in fixing the α-carboxylic group of the substrate (or any structurally equivalent group from other ligands), which leads to the correct positioning of the α-amino group in close proximity to the PLP C4' catalytic center. Moreover, when a substrate or inhibitor molecule occupies the active site, the Tyr101 side chain undergoes a drastic repositioning to make room for the benzyl or indole group, respectively, while the presence of glycerol or tris(hydroxymethyl)aminomethane molecules in the active site (PDB entries: 3FVS; 3FVX) does not promote such a rearrangement (Han et al., 2009b). These studies confirm Trp18 in the N-terminal arm as a key residue for substrate/inhibitor binding to the active site. In fact, by approaching the ligand molecule, Trp18 completes the formation of the narrow, mainly aromatic ligand binding cavity and constrains sliding of the short region of the N-terminal arm (residues 14–34), which shields the active site during catalysis. Interestingly, in the structure of hKAT I in complex with I3AA, two ligand molecules (I3AA and glycerol) are simultaneously present in each active site. This observation allowed the authors to propose a possible pathway acting as the exit for KYNA at the end of the first half-reaction and/or for the incoming α-ketoacid that initiates the second half-reaction (Han et al., 2009b), shedding light on a still elusive aspect of KAT-mediated catalysis, that is, the series of events that brings the enzyme back to its starting state, ready for a new catalytic cycle.

As expected from the high sequence identity between hKAT I and mKAT III (49.8%) (Yu et al., 2006), comparison of their crystal structures confirmed that the two isozymes share the same fold and a similar mode of subunit assembly in the form of a functional dimer, while they differ in a few residues that build up the walls of the ligand-binding cavity. These peculiarities could represent the basis for the different substrate specificities and catalytic properties displayed by the two isozymes (Han et al., 2009a). Most recently, the original crystal structures describing mKAT III in PLP-bound form (original PDB entry: 3E2F), in complex either with L-KYN and PMP (original PDB entry: 3E2Z), or glutamine and PMP (original PDB entry: 3E2Y) (Han et al., 2009a) underwent a re-refinement process (Wlodawer et al., 2018). This new analysis suggests that the electron density signals initially assigned to two glycerol molecules, or to a molecule of KYN or glutamine non-covalently bound at the active site, should instead be interpreted as HEPES molecules, which were present in the crystallization buffer (Han et al., 2018). Notably, the observation of HEPES molecules in the re-refined structures highlights the possibility for the ligand to lay in two alternative sub-cavities in the ligand binding site (Figure 2C). In the PLP-bound holo-form of mKAT III (re-refined PDB entry: 5VEP), the ligand molecule occupies a narrow, elongated pocket underneath the region of the N-terminal arm spanning from Asp50-Trp54. In both the PMP-bound structures (re-refined PDB entries: 5VEQ and 5VER), the HEPES molecule appears to be rotated 45° toward the previously identified “canonical” ligand-binding cavity, keeping the sulfate group in contact with Arg430. The shift between the two ligand conformations seems to be facilitated by repositioning of the Trp54 side chain. Although serendipitously discovered, this information could be used for the better understanding of the mechanistic aspects and molecular dynamics of KAT-mediated catalysis.

The crystal structure of mKAT IV has been solved in its PLP-bound form (PDB entry: 3HLM), in complex with the amino group donor/substrate kynurenine, or with the amino group acceptor/co-substrate oxaloacetate (PDB entries: 3PD6 and 3PDB, respectively) (Han et al., 2011). These structures reveal an overall architecture that closely resembles the one described for mitochondrial aspartate aminotransferases from other species (Han et al., 2010b). Similarly, substrate binding does not appear to be accompanied by a significant reshaping of the catalytic cavity; instead, each monomer switches from an “open” to a “closed” form as a consequence of rotation of the small domain toward the large domain (Ford et al., 1980; McPhalen et al., 1992).

From a structural standpoint, KAT II is a “maverick” among the four KATs, and its uniqueness is made clear by comparing the numerous crystal structures of hKAT II in different ligand-bound states that are currently present in the Protein Data Bank. This wealth of information is the result of recent efforts aimed at the selection and/or optimization of small molecules acting as KYNA synthesis attenuators, that is, molecules capable of lowering KYNA levels in pathological conditions without completely suppressing KYNA-dependent neuroprotective actions. Since KAT II is thought to be the main factor responsible for KYNA synthesis in the mammalian brain (Guidetti et al., 1997; Schwarcz et al., 2012; Chang et al., 2018), and considering that the peculiar KAT II characteristics make it unique among the functionally validated KATs (see below), the rational design of isozyme-specific inhibitors appeared feasible and indeed was successful [comprehensive reviews on progress in KP pharmacological manipulation and in the development of KAT inhibitors appear in Dounay et al. (2015), Zádori et al. (2016), and Nematollahi et al. (2016b)].

The most striking feature of the hKAT II structure is the swapped conformation adopted by a discrete region of the N-terminal arm of each subunit (residues 16–39) (Figure 2D). This region acts as a dynamic element that simultaneously shields and shapes the ligand binding cavity of the other monomer in the dimer to an extent that depends on the nature of the bound molecule. The functional role of the intrinsic conformational plasticity displayed by the swapped region of hKAT II can be fully appreciated upon superposition of the entire panel of protein crystal structures -in complex with PLP (PDB entries: 2VGZ, 2QRL, 5EUN) (Han et al., 2008b; Rossi et al., 2008a; Nematollahi et al., 2016a), substrates (PDB entries: 3DC1, 2R2N) (Han et al., 2008a,b) or inhibitors (PDB entries: 2XH1, 3UE8, 4GE4, 4GE7, 4GE9, 4GEB) (Rossi et al., 2010; Dounay et al., 2012, 2013; Tuttle et al., 2012). As observed in the other KAT structures, anchoring of the α-carboxylic group of the physiological substrate (or of any structurally equivalent group from the inhibitor molecule) is invariably provided by Arg399 regardless of whether there is a covalent bond between the ligand and cofactor. The noteworthy broad ligand binding potential of hKAT II seems to rely on the chemical nature of the residues lining the catalytic cavity and, most importantly, the possibility to modulate the available space and protein/ligand bonding networks, which largely depends on the dynamics of the swapped region. This is clearly exemplified by the evolution of compounds **1** and **2** in Figure 2D, which are the most potent isozyme-specific brain penetrant inhibitors of hKAT II currently available (Tuttle et al., 2012; Dounay et al., 2013).

The structural information on KATs summarized here strengthens the initially hypothesized fundamental role of a discrete region of the N-terminal arm of KATs in controlling substrate/inhibitor access into and binding inside the active site (Rossi et al., 2008b). In hKAT I and mKAT III, the equivalent regions slide toward the preformed catalytic cavities as rigid bodies, which places the invariant Trp18 or Trp54 residues in an optimal position to entrap the ligand. In hKAT II, the same region is characterized by a higher degree of conformational freedom, which is apparently required to better adapt to a broader array of ligands that differ in size, chemical nature and steric hindrance, thus resulting in a more pronounced reshaping of the active site. However, both situations highlight the need for anchoring points at both sides of the region of the N-terminal arm that moves (Figure 2D) to limit its unrestrained flexibility. Notably, a *kat1* gene variant isolated from spontaneously hypertensive rats (SHR) is characterized by a missense mutation that leads to a Glu-to-Gly amino acid substitution at the C-terminal side of the sliding α-helix in the KAT I N-terminal arm (Kwok et al., 2002). The elimination of this “distal lock” could hamper proper active site shielding upon substrate binding and ultimately translate into suboptimal catalysis.

## Future Directions for KAT Structural Investigations

The analysis of KYNA synthesis from an integrated structural biology/neuropharmacology standpoint will drive the lead optimization process aimed at further improving the pharmacodynamics, bioavailability and specificity of action for the most potent hKAT II inhibitors developed thus far, as a prerequisite to their safe use in pathological situations characterized by abnormally elevated brain KYNA levels. However, due to the potential adverse effects associated with the irreversible inhibition of KATs, the future challenge for the field is the identification and/or the structure-based design of molecules capable of reversibly associating with specific KAT targets (Nematollahi et al., 2016b). Among the different compounds that behave as competitive KAT ligands, one potential source of such innovative hKAT II-specific reversible inhibitors is a recently described group of sulfated oestrogens and their derivatives (Jayawickrama et al., 2017, 2018).

At the same time, by revealing the determinants of substrate specificity in specific KAT isozymes, a combined structural biology/biochemistry approach could provide new small molecule tools to better characterize the interplay between the alternative branches of the KP in physiological conditions and in human diseases. A paradigmatic example of the applicability of such an approach is its recent use to study the roles of XA in the CNS. In light of the pro-apoptotic and neurotoxic potential associated with 3-HK (Okuda et al., 1998), it is somewhat surprising that the KAT-dependent transformation of 3-HK to XA had not been analyzed at the same level of detail as the transamination of KYN to KYNA, although the label “KAT” explicitly tags the XA-producing reaction in schematic representations of the KP. Once considered a mere by-product of the KP, research on XA came back in focus only recently following the description of its roles in invertebrate biology (Savvateeva et al., 2000; Cerstiaens et al., 2003). In most insect species, the central KP branch abruptly ends with the synthesis of the free radical generator 3-HK. The production of XA is therefore a way to protect the organism from the accumulation of 3-HK when it is not required for the biosynthesis of eye and body pigments or tissue remodeling during (neuro)metamorphosis (Han et al., 2007). Notably, in haematophagous insects, XA acts as a scavenger for free iron derived from *heme* demolition, and XA deficiency has been associated with direct oxidative damage to insect midgut epithelial cells (Lima et al., 2012). Moreover, XA produced by *Anopheles gambiae* 3-hydroxykynurenine transaminase (Rossi et al., 2005, 2006) is a trigger for *Plasmodium* male gametogenesis, which takes place in the mosquito upon a parasite-infected blood meal (Billker et al., 1998). XA also exerts multiple actions in the mammalian brain (Sathyasaikumar et al., 2017; Schwarcz and Stone, 2017). Of particular significance is the observation that levels of XA are reduced in the brain and serum of schizophrenic patients, which is opposite to what has been reported for KYNA (Fazio et al., 2015). By using different approaches, including *in vivo* analyses, it has been proposed that KAT II could be the main determinant of XA synthesis in the CNS (Sathyasaikumar et al., 2017). Although XA and KYNA production appears to be sustained by distinct brain cell populations (Roussel et al., 2016), these studies highlight the need to analyse the whole complement of KAT isozymes for their role in XA synthesis and to re-evaluate the impact of specific KAT inhibitors on the local balance of these two fundamental products of the KP.

## Author Contributions

All authors listed have made a substantial, direct and intellectual contribution to the work, and approved it for publication.

### Conflict of Interest Statement

The authors declare that the research was conducted in the absence of any commercial or financial relationships that could be construed as a potential conflict of interest.
